# Conditioned Pain Modulation Is Associated with Common Polymorphisms in the Serotonin Transporter Gene

**DOI:** 10.1371/journal.pone.0018252

**Published:** 2011-03-28

**Authors:** Fredrik Lindstedt, Jonathan Berrebi, Erik Greayer, Tina B. Lonsdorf, Martin Schalling, Martin Ingvar, Eva Kosek

**Affiliations:** 1 Department of Clinical Neuroscience, Osher Center for Integrative Medicine, Stockholm Brain Institute, Karolinska Institutet, Stockholm, Sweden; 2 Department of Systems Neuroscience, University Hospital Eppendorf, Hamburg, Germany; 3 Neurogenetics Unit, Department of Molecular Medicine and Surgery and Center for Molecular Medicine, Karolinska Institutet, Stockholm, Sweden; University of Chicago, United States of America

## Abstract

**Background:**

Variation in the serotonin transporter (5-HTT) gene (*SLC6A4*) has been shown to influence a wide range of affective processes. Low 5-HTT gene-expression has also been suggested to increase the risk of chronic pain. Conditioned pain modulation (CPM) - i.e. ‘pain inhibits pain’ - is impaired in chronic pain states and, reciprocally, aberrations of CPM may predict the development of chronic pain. Therefore we hypothesized that a common variation in the *SLC6A4* is associated with inter-individual variation in CPM. Forty-five healthy subjects recruited on the basis of tri-allelic 5-HTTLPR genotype, with inferred high or low 5-HTT-expression, were included in a double-blind study. A submaximal-effort tourniquet test was used to provide a standardized degree of conditioning ischemic pain. Individualized noxious heat and pressure pain thresholds (PPTs) were used as subjective test-modalities and the nociceptive flexion reflex (NFR) was used to provide an objective neurophysiological window into spinal processing.

**Results:**

The low, as compared to the high, 5-HTT-expressing group exhibited significantly reduced CPM-mediated pain inhibition for PPTs (p = 0.02) and heat-pain (p = 0.02). The CPM-mediated inhibition of the NFR, gauged by increases in NFR-threshold, did not differ significantly between groups (p = 0.75). Inhibition of PPTs and heat-pain were correlated (Spearman’s rho = 0.35, p = 0.02), whereas the NFR-threshold increase was not significantly correlated with degree of inhibition of these subjectively reported modalities.

**Conclusions:**

Our results demonstrate the involvement of the tri-allelic 5-HTTLPR genotype in explaining clinically relevant inter-individual differences in pain perception and regulation. Our results also illustrate that shifts in NFR-thresholds do not necessarily correlate to the modulation of experienced pain. We discuss various possible mechanisms underlying these findings and suggest a role of regulation of 5-HT receptors along the neuraxis as a function of differential 5-HTT-expression.

## Introduction

Chronic pain poses a major clinical challenge, markedly reducing quality of life for many individuals as well as imposing a high socioeconomic burden on society [1], [2]. The familial aggregation of chronic pain syndromes suggests that the risk for developing pathological pain is strongly influenced by genetics [3], [4]. Investigations into central processes underlying pain perception has provided evidence for impairments of regulatory systems, e.g. impaired pain-inhibition, as a potentially important factor in the development of persistent pain [5]. Therefore, studies aimed at teasing out the genetic underpinnings underlying such individual differences in pain regulation may help to identify clinically relevant biomarkers.

Extensive electrophysiological studies in rodents during heterotopic noxious conditioning stimulation (HNCS) revealed a potent, widespread and selective inhibition of wide dynamic range (WDR) neurons in the dorsal horn and trigeminal nuclei. These mechanisms were coined ‘diffuse noxious inhibitory controls’ (DNIC) by Le Bars and colleagues [6], [7], [8] and rely on descending projections from neurons with whole-body receptive fields in the subnucleus reticularis dorsalis (SRD) in the caudal brainstem [9]. It was subsequently shown that DNIC-like effects induced by thermal conditioning stimuli are present in humans, i.e. ‘pain inhibits pain’, and that these could be assessed using the nociceptive flexion reflex (NFR) [10]. Studies of DNIC-like effects in humans also use reports of the subjective experience, consequently capturing effects of uncertain neurophysiologic origin. It has therefore recently been recommended that the collective phenomena should be referred to as conditioned pain modulation (CPM) rather than ‘DNIC’ when studied in humans [11]. In this paper we will adopt the new terminology.

CPM-studies have revealed dysfunctional pain regulation in patients with various types of chronic pain, e.g. fibromyalgia [12] and painful osteoarthritis [13]. Additionally, altered CPM-responses have been reported in tension-type headache and migraine [14] as well as in atypical trigeminal neuralgia [15]. Importantly, individual CPM-response has in a prospective design been tied to the risk of currently healthy individuals developing chronic postsurgical pain [16].

The initial DNIC-studies in rodents suggested a pivotal role of descending serotonergic projections. Whereas administration of the serotonin (5-HT) precursor 5-hydroxytryptophan (5-HTP) led to a more potent inhibition, the 5-HT receptor antagonist cinanserin reversed this potentiation [17], [18]. A key player in 5-HT signaling is the 5-HT transporter (5-HTT), terminating the extracellular effects of 5-HT by sodium-dependent intracellular re-uptake [19]. Drugs acting on this integral membrane protein have a place in the pharmacological arsenal used against unrelenting pain. Although selective serotonin re-uptake inhibitors (SSRIs) play a limited role in pain treatment, serotonin-noradrenalin re-uptake inhibitors (SNRIs) and tricyclic antidepressants are commonly used to treat various forms of chronic pain. The exact mechanisms dictating whether these classes of drugs are successful in any particular patient are poorly understood [20]. As both pain and analgesic responses have large hereditary components [21], a genetic approach in mechanistic pain studies may both lead to an increased understanding of the pathogenesis of chronic pain as well as suggest pharmacogenomic options for improving treatment.

The human 5-HTT is coded for by a single gene (*SLC6A4*) residing on the long arm of chromosome 17 [22]. Polymorphisms in the promoter region of *SLC6A4* are some of the most well-studied sources of variation in psychiatric genetic research [23]. The initial impetus for such studies came from a report in 1996 that a repeat length polymorphism, the so-called 5-HTT linked polymorphic region (5-HTTLPR), located in the promoter region of the *SLC6A4* affects transcriptional efficiency. The 5-HTTLPR is a 43 base pair insertion/deletion in a C/G-rich variable number tandem repeat (VNTR) sequence located in the promoter region, yielding a long (l) and a short allele (s) [24]. The s-allele is coupled to a reduced gene-expression, leading to lower densities of 5-HTT receptors, and has been implicated in a wide variety of anxiety disorders and depression [25] and also associated with pain states such as migraine [26] and fibromyalgia [27], [28].

The *SLC6A4* harbors many other polymorphisms in addition to the 5-HTTLPR. In the same promoter region, a single nucleotide polymorphism (rs25531), implying an A to G substitution, has been further shown to alter the degree of gene expression [29]. The minor G-allele is nearly always in phase with the 5-HTTLPR l-allele and is suggested to reduce the transcriptional efficacy to levels similar to the s-allele. When studied jointly the 5-HTTLPR/rs25531 mini-haplotype is usually referred to as ‘tri-allelic’ 5-HTTLPR (due to the very low frequency of the fourth allele, S_G_, this is often ignored in studies) implying the functional division of individuals into high (L_A_/L_A_), intermediate (L_A_/L_G_, S_A_/L_A_) or low (S_A_/S_A_, L_G_/S_A_) 5-HTT-expression types respectively [30]. Ethnic differences are reported and allelic frequencies differ within European populations [31]; tri-allelic frequencies are reported to be around 43% (S_A_), 6.5% (L_G_) and 50% (L_A_) [30].

The aim of the present study was to assess whether individual CPM-response is associated with the tri-allelic 5-HTTLPR. Given the reported findings of lower 5-HTT gene-expression in chronic pain [27], [32], we hypothesized that the low 5-HTT-expressing group would exhibit a lesser degree of CPM-mediated pain inhibition. We employed an individually titrated conditioning stimulus, namely the submaximal effort tourniquet test (SETT) [33], and applied three different types of test-stimuli. CPM was thus studied on individually calibrated supra-threshold noxious heat, pressure pain thresholds (PPTs) and the nociceptive flexion reflex (NFR). The NFR is an established electrophysiological measure of spinal nociceptive processing [34], [35]. To our knowledge this is the first study assessing the effects of CPM on the basis of tri-allelic 5-HTTLPR. Further it is, to the best of our knowledge, the first report of using the NFR as an ‘objective’ modality together with other, subjective and qualitatively separate, modalities of test-stimuli within the same CPM-session.

## Methods

### Participants

A total of 45 healthy volunteers of European descent, pre-selected on the basis of their tri-allelic 5-HTTLPR genotype, were included in the study. The low 5-HTT-expressing group (S_A_/S_A_, L_G_/S_A_) contained 22 individuals (13 females). The high 5-HTT group (L_A_/L_A_) was comprised of 23 individuals (15 females). See Table 1. One additional subject initially enrolled was excluded from further analysis because of reporting current chronic pain problems during the post-experimental debriefing.

**Table 1:** Participants.

	N total	N female	N male	Median age	Age range
Low 5-HTT-expressing group	22	13	9	25.5	20–52
High 5-HTT-expressing group	23	15	8	25	20–54

Healthy subjects were recruited based on genotype. Both subjects and experimenters were blinded for genotype. The members of the groups did not differ significantly in age [U = 239.5, z = −0.31, p = 0.76] and women did not differ significantly in their menstrual cycles between groups [χ^2^(1) = 0.001, p = 0.98].

The study was conducted according to the principles expressed in the Declaration of Helsinki and was approved by the Regional Ethical Review Board in Stockholm (reference number 2010/716 – 32). All participants gave their written informed consent. Subjects were paid for their participation.

Subjects were recruited on the basis of tri-allelic 5-HTTLPR genotype from a pool of approximately 500 individuals. Both the experimenters and volunteers were blinded for the genotype. DNA-samples came from volunteers at a variety of institutions, in the Stockholm area. Individuals in the pool had provided informed consent for DNA-analysis and agreed to be contacted for invitations to participate in research projects within the neurosciences. Subjects in the pool were naïve to our paradigm and had not participated in any similar experiments conducted by our group. To meet the inclusion criteria participants had to be healthy, non-pregnant, adults without pain problems and not suffer from any present or previous psychiatric disorder. Subjects were of European descent. Except for contraceptives, subjects were not included if they were currently using any pharmaceuticals that could potentially interact with pain perception. These factors were initially assessed by a brief phone interview during the recruitment process and further confirmed on the day of testing. The reported experiment was the last in a battery of much less invasive sensory tests, conducted on the same day in an identical manner for all recruited subjects (data will be reported elsewhere). The other experiments consisted of assessing non-noxious and threshold temperatures against the ventral forearm, measurements of the acoustic startle reflex and a trial of cognitive modulation of pain. In the latter noxious heat (<49°C) was applied to the left ventral forearm, five times for 30 seconds each time, with an interval of 5 minutes between each stimulation. Subjects were given a 10 minute break before the start of the present experiment.

### Genotyping

Samples for DNA-extraction were either obtained in the form of 20 ml whole blood or saliva. DNA-extraction from whole blood was performed as described earlier [36] and from saliva using the protocol and reagents in the Oragene® kit (DNA Genotek Inc, Kanata, Canada).

To determine the tri-allelic 5-HTTLPR, PCR-reactions were carried out in a total volume of 20 µl containing 50 ng of genomic template, 0.2 nM of each dNTP, 1 mM of each primer (Thermo Scientific, Ulm, Germany), 0.05 U/µl Quiagen HotStar®Polymerase, 1 M Q-solution and 1x Buffer. The forward primer sequence was 5’-GGCGTTGCCGCTCTGAATGC-3’ and the reverse 5’-GAGGGACTGAGCTGGACAACCAC-3’. Samples were amplified (Biorad Tetrade, Hercules, CA,USA), following an initial denaturation step for 10 min at 94°C, by 32 cycles of 30 s denaturation (95°C), annealing for 30 s (57°C) and elongation for 30 s (72°C). This was followed by a final elongation step for 5 min at 72°C. The described PCR yields a long (529 bp) and a short (486 bp) fragment which were visualized with UV after 2 h separation at 180 V on a 2.5% agarose gel containing GelRed®. Additionally, 10 µl of the PCR product were digested for 12 h at 37°C with 0.1 µl MSP1 (New England Biolabs, Ipswitch, MA, USA) and 1 µl buffer per sample. The MSP1 cuts at a 5’-C/CGG-3’ sequence. This results in fragments of different length from which the tri-allelic 5-HTTLPR genotype can be determined. L_A_ results in 340 bp, 127 bp and 62 bp; S_ A_ results in 297 bp, 127 bp, and 62 bp; L_ g_ results in 174 bp, 166 bp, 127 and 62 bp; S_ g_ (very uncommon) results in 166 bp, 131 bp,127 bp and 62 bp. Samples with the thus digested fragments were visualized using UV-light after being run for 2 h at 180 V on 4% agarose gels containing GelRed®

Using the available samples we thus unambiguously genotyped 94% ( = call-rate) of the individuals (478 of 511) in our database. The genotyped individuals in the database did not differ significantly from Hardy-Weinberg equilibrium (HWE) with regard to the bi-allelic 5-HTTLPR [χ^2^(1) = 2.9, p = 0.1] or the rs25531 [χ^2^(1) = 1.5, p = 0.2], used to construct the tri-allelic 5-HTTLPR. As mentioned, subjects included in the actual experiment were selected on the basis of this genotype, rendering any HWE-calculations for the distribution in the present study irrelevant.

### Questionnaires and scales

Prior to testing, subjects completed the state-part of a Swedish version of the State Trait Anxiety Inventory (STAI) - questionnaire. After the main experiment, subjects completed the trait-part of the STAI as well as a Swedish version of Beck Depression Inventory (BDI). To increase the participants feeling of integrity, they were provided with envelopes for the questionnaires. If any part of a questionnaire was left blank, multiple answers were chosen or answers marked ambiguously, the questionnaire was excluded from analysis.

One-hundred millimeter long visual analog scales (VAS) were used for assessment of heat-pain. The left hand side was labeled ‘no pain’ and the right hand side ‘worst pain imaginable.’ Scales were printed on separate sheets of paper, a new one used for each rating. For verbal ratings of ischemic pain a Borg CR10 scale was used [37]. This ties standardized descriptors to corresponding numerical ratings ranging from 0 to 11 (‘worst possible pain’).

### Experimental protocol, an overview

All testing was conducted during daytime to mitigate the influence of circadian factors on pain perception. Testing was performed by the same lead and assistant experimenters and subjects were given ritualized instructions. Upon arrival at the experimental facility, volunteers provided written informed consent.

Subject sat comfortably upright in a 3-sectioned clinical examination bed and the individual temperature to be used for heat-pain testing was calibrated. Electrodes for NFR-measurements were fitted on the right foot and leg and an uninflated blood- pressure tourniquet placed around the upper left arm. Firstly, a baseline reading of pressure pain threshold (PPT) was assessed using algometry at the right masseter muscle. Secondly, baseline VAS-ratings were obtained during a 30-second long heat-stimulus. An individualized level of noxious heat was applied to the skin overlying the right quadriceps muscle and VAS-ratings were provided halfway through (i.e. 15 seconds) and at the end of the stimulus (i.e. 30 seconds). Thirdly, the threshold level of the NFR was assessed twice. The conditioning stimulus was then induced using a submaximal-effort tourniquet test (SETT) as described below and titrated individually to a pain rating of 6 on the Borg CR10 scale (range 0-11). When the SETT-pain rating had reached 6 (or 60 grips had been conducted) the CPM-test thus commenced. To assess conditioned pain modulation (CPM), test-stimuli were applied in their initial order during the concurrent ischemic conditioning pain provided by the SETT. The PPT was assessed immediately at the start of the CPM-test. One minute into the CPM-test, noxious heat was applied to the right quadriceps and rated on VAS-scales as during baseline. Two minutes into the CPM-test, measurement of NFR-threshold started. Subjects also rated the ischemic pain intensity during the CPM-test at the one and two minute marks. Details are provided below.

### Heat-pain ratings and temperature calibration

A computer controlled Peltier-type thermode with a 30 mm×30 mm surface was used for all thermal testing (PATHWAY model ATS, Medoc, Israel). An initial calibration was performed to individualize the temperature to be used for the heat-pain test-stimulus. Whereas the skin overlying the right quadriceps was used for actual CPM-testing, the right ventral forearm arm was used for this calibration in order to avoid sensitization of the leg. A total of six 15 second stimuli, with an end-to-onset interstimulus interval of 30 seconds, were applied starting from a baseline of 32.0°C. Destination and return rates were set at 10.0°C/s. Temperatures of 46.0°C, 47.0°C and 48.0°C, with two trials of each, were applied in a counterbalanced and double-blinded order. VAS-ratings in mm, provided as soon as the temperature dropped back to baseline, were entered into a linear regression to determine the temperature corresponding to approximately a 60 mm VAS-rating. For safety reasons, the maximum temperature was set to 48.9°C.

### Baseline pressure pain thresholds (PPTs)

The PPT is defined as the lowest pressure (measured in kPa) that, using standardized testing conditions, needs to be applied in order to cause the slightest sensation of pain. It is a reliable and widely used measure [38]. PPTs were measured with an algometer (Somedic Sales AB, Hörby, Sweden) fitted with a 1 cm^2^ pressure probe and calibrated using a manufacturer-supplied weight corresponding to 100 kPa. Algometry was performed over the belly of the right masseter muscle with an approximate rate of pressure increase of 50 kPa/s. To acquaint subjects with the procedure and improve their accuracy on reporting the ‘slightest pain’ by means of pressing a button, an initial trial was conducted accompanied with verbal instructions. This was immediately followed by the real test. Testing during conditioning SETT-induced pain was identical.

### Baseline heat-pain

The individualized temperature for noxious heat was applied to the skin overlying the right quadriceps femoris muscle, approximately 5 cm proximal to the patella. The temperature was applied for 30 seconds using the experimenter-held thermode. From a baseline of 32.0°C the temperature increased with 10.0°C/s. Pain was rated at 15 seconds and at the end of the stimulus. Testing during conditioning SETT-induced pain was identical.

### NFR-threshold determination

#### Apparatus and NFR-thresholding program

The skin overlying the right sural nerve was cleaned and abraded using prepping-paper (3M Red Dot Trace, Cephalon, Nørresundby, Denmark). A disposable dual electrode with 20 mm center-to-center distance (Viasys nr 019-429400, Cephalon) was placed in the retromalleolar fossa on the skin overlying the path of the sural nerve. Before placement approximately 0.1 ml of salt-free electrode gel (Spectra® 360, Parker Laboratories Inc, Fairfield, New Jersey, USA) was applied to each of the two foam pads using a syringe. The dual electrode was connected to snapleads (Viasys nr 019-424500, Cephalon) with the cathode placed proximally. The impedance between the attached electrodes was verified to be less than 10 kOhm using a UFI checktrode model 1089e (UFI, California, USA).

The right biceps femoris muscle was used for electromyographic (EMG) measurements and electrodes were placed approximately 10 cm superior to the popliteal fossa and halfway between the lateral aspect and the midline of the leg. The area was shaved if needed and thereafter cleaned and abraded using prepping-paper. Disposable dry foam electrodes (EL509, BIOPAC Systems Inc, Goleta, California, USA) were filled with salt-free electrode gel according to the manufacturer’s instructions and juxtapositioned on the site. An identical electrode for grounding was placed over the right proximal fibula. Shielded leads were connected to the biceps femoris electrodes and connected to an EMG unit (EMG100C and MP150, BIOPAC Systems Inc) with gain set at 5000, 500 Hz low-pass, and 10 Hz high-pass filtering and a sampling rate of 2 kHz. For stimulation output to the subject, an optically isolated constant current stimulator was used (STMISOL, BIOPAC Systems Inc). The stimulation curve was fed to the stimulator using a data acquisition system (USB 6221 M DAQ Module, National Instruments Corporation, Austin, Texas, USA) connected to a computer. The stimulation signal was reliably synchronized to the EMG-signal.

Each shock consisted of a train of 5 identical square wave pulses, each 1 ms in duration, spaced 3 ms apart. A commonly used staircase algorithm [39], increasing and decreasing the stimulus level in steps of 4 mA, 2 mA and finally 1 mA, was implemented in a MATLAB-program to find the NFR-threshold. The time between two shock trains was randomized and gave a minimum of 7 seconds and an average of 10 seconds. This inter-stimulus interval was chosen to minimize the risk of sensitization or habituation phenomena and at the same time make the total measurement time short enough to allow testing during the conditioning pain procedure.

Our program used the detection rule suggested by France and co-workers based on the interval-mean of the EMG-signal [39], [40]. The EMG-signal was rectified and the interval-mean of the baseline from 0 ms to 65 ms prior to stimulus onset was compared to the interval-mean of the rectified signal 90 ms to 150 ms after the last pulse of the shock-train by means of a z-score. This post-stimulus time window allows assessment of the RIII-component of the flexion reflex, without contamination of the earlier RII-component [39]. An NFR was reported to have occurred for interval mean z-scores >1.4.

The experimenter validated the program’s interpretation (i.e ‘NFR detected’ or ‘no NFR’) using an online graphic display of the EMG from the timeframe surrounding the last stimulation. In case of e.g. excessive muscle movement in the pre-stimulus baseline, as evident in the EMG-signal, the experimenter would consequently choose to repeat the stimulus at the same current strength. Otherwise, by default, the program continued to the next stimulus level as suggested by the algorithm until the threshold had been calculated.

#### NFR-threshold measurement

All subjects wore shorts during testing and were seated comfortably in a 3-sectioned neurophysiological examination bed (Sjöbloms Sjukvårdsutrustning AB, Örnsköldsvik, Sweden). A cylindrical cushion, with 20 cm diameter, was placed below the right knee and the leg section lowered to give a 120° flexion at the knee. Subjects were instructed to sit as relaxed as possible with their eyes open, looking straight ahead. A few test stimuli of 2 mA, applied to acquaint subjects with the sensation, were followed by two consecutive baseline threshold calibrations. In assessing the threshold, subjects were not asked to rate the pain from the shocks as this could have confounded the measurement in the light of the evidence of anticipatory effects on reflex activity [41]; we wanted to use the NFR as a purely neurophysiologic measurement. Measurement of the NFR-threshold during conditioning SETT-induced pain was identical.

### Submaximal effort tourniquet test (SETT) and CPM-testing

Subjects were told that the measurements conducted during baseline would be repeated during the SETT but were not informed about the specific purpose of the test or of the expected results. A blood-pressure tourniquet (TriCUFF® original, AJ Medical, Stockholm, Sweden) was applied to the bare upper left arm. After having the baseline values determined for PPT, heat-pain and NFR-threshold as described above, the SETT commenced. Subjects carried out three maximal-effort isometric grip exercises, using a grip-training device (Jym™, Jym Fitness, Kingswood, Australia), with their left hand. The highest value was recorded and the device programmed to give emit a beep whenever more than 50% of this force was applied.

Subjects then elevated their left arm for approximately 2 minutes for partial exsanguination whereupon the blood pressure cuff was inflated to 250–260 mmHg. To attenuate individual factors relating to the possibly unpleasant visual stimulus of a discolored extremity, all subjects were fitted with a green sleeve over the arm and hand. Using their ischemic arm, subjects then carried out grip exercises with the Jym™ device, releasing the pressure as soon as half of their maximal grip strength was reached (indicated by a beep). Grips were synchronized with a metronome and conducted at 3 second intervals. Every 5 grips participants verbally rated the arm pain with numbers chosen from a Borg CR10 scale until a level of 6 or above was reached or until a total of 60 grip-exercises had been performed, whichever came first. This marked the start of the CPM-testing, during which the tourniquet remained inflated between 250 mmHg and 260 mmHg. Importantly, subjects were not told beforehand when the gripping would be suspended.

### Statistics

SPSS Statistics 17.0 (SPSS Inc, Chicago, USA) was used for all analyses. Two-tailed tests were used unless otherwise stated. P-values<0.05 were considered significant but Bonferroni-adjusted to control for multiple comparisons where stated. Data is reported as means ±1 standard deviation (SD) and graphs as means with error bars of ±1 standard error of the mean (SEM).

**Figure 1:**
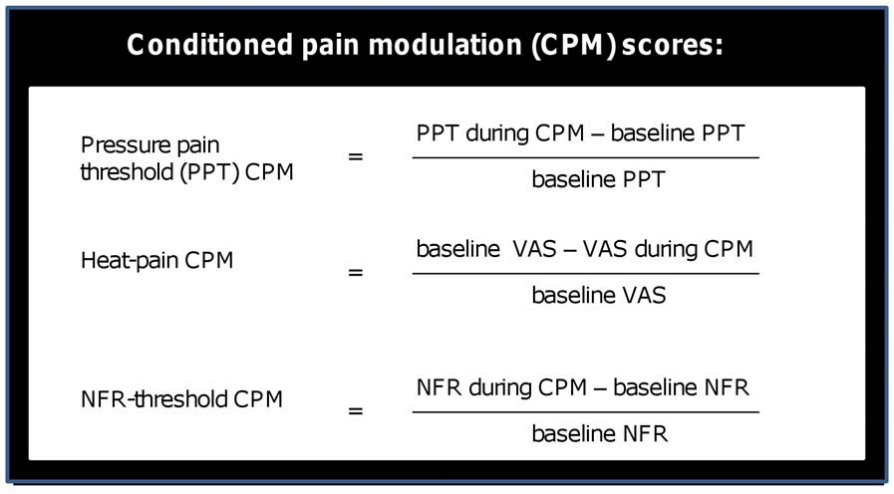
Conditioned pain modulation scores (CPM-scores). To control for individual variation with regard to baseline values, the proportion of difference from baseline was used rather than the difference of raw values. This was done to control for individual variation in baseline measures and gives positive CPM-scores for pain inhibition and negative ones for facilitation, as compared to the baseline. To enable a comparison of VAS-ratings with threshold values, we define our CPM-scores as q(b–c)/b where b = baseline value (in kPA, mm or mA) and c =  value during CPM. q = 1 for heat-pain VAS-ratings and q = −1 for thresholds ratings.

To calculate the effects of the conditioning pain modulation (CPM), individual CPM-scores were calculated. See Figure 1. For these, the proportion of difference from baseline was used rather than the raw difference, in order to control for individual variation in baseline measures. This gives positive CPM-score values for pain inhibition and negative ones for facilitation, as compared to the baseline.

Raw data and derived measures were analyzed with Shapiro-Wilk tests to assess significant deviations from normality. Independent t-tests were used to analyze differences between the genotype groups for STAI-data. A paired-sample t-test was used to validate the effect of the SETT on the NFR-threshold. Univariate analyses of variance, with genotype and gender as fixed factors, were used for analyzing PPT CPM-scores, baseline heat-pain, average baseline NFR-thresholds, NFR-threshold CPM-scores and the number of grips needed during the SETT-procedure. Heat-pain VAS-ratings for 46.0°C, 47.0°C and 48.0°C (each applied twice in a counterbalanced order) were averaged for each temperature for each subject and entered into a factorial repeated-measures ANOVA, with gender and genotype as between-subject factors. Greenhouse-Geisser corrected degrees of freedom were used for this ANOVA as the assumption of sphericity was violated.

Non-parametric tests were used whenever the assumption of normality was violated. Mann-Whitney U tests (exact) were used for comparing genotypes for age, BDI-data, baseline PPTs, individualized temperatures used for test-stimuli, CPM-scores for heat- pain, ischemic pain ratings and grip-strength during the SETT. To assess the potential influence of menstrual cycle, females were dichotomized by luteal- and follicular-phases. A Pearson χ^2^-test was used to check for differences in these menstrual cycle-phases between genotype groups. Due to the constrained sample size while only studying females, Mann-Whitney U tests (exact) were used in assessing the relation between menstrual cycle-phase and CPM-scores. Wilcoxon signed-rank tests (exact) were used as a manipulation-check for the effect of the SETT on PPTs and to test for habituation or sensitization between 15 s and 30 s baseline VAS-ratings for heat-pain.

## Results

### Questionnaires

For the Beck-depression inventory (BDI), 1 subject’s questionnaire was excluded. Three subjects in each gene group were excluded for the state-anxiety and a total of 3 were excluded for the trait-anxiety parts of the STAI-questionnaires. No differences between the genotype groups were found for BDI-score [U = 228, z = −0.32, p = 0.76] or trait-anxiety [t(40) = 0.35, p = 0.73]. However, the low 5-HTT-expressing group reported a significantly [t(37) = 2.05, p<0.05] higher state-anxiety (mean 31.5±6.9) compared to the high 5-HTT-expressing group (mean 27.5±5.3).

### Pressure pain thresholds (PPTs): baseline and during SETT-pain

**Figure 2:**
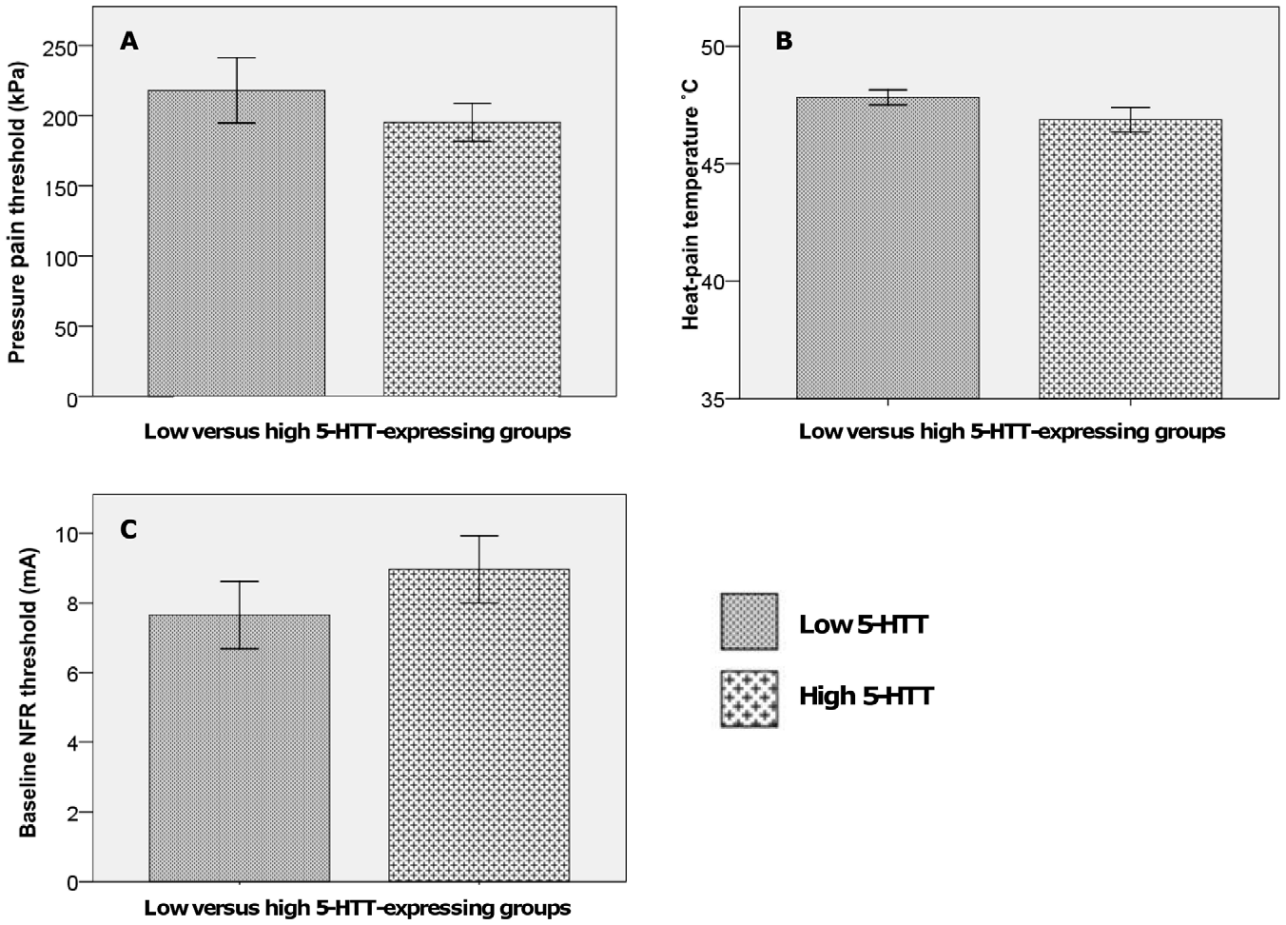
Baseline values for test-pain stimuli. A. Pressure pain thresholds (PPTs) in kPA, assessed by algometry, at baseline. Genotype did not have a significant effect on the baseline level [U = 247, z = −0.14, p = 0.90]. B. No significant differences were found on the basis of genotype [U = 211, z = −0.95, p = 0.35] between the individualized temperatures used as test-stimuli. C. Average of the two baseline NFR-threshold measurements in mA. No significant differences were found between groups, F<1.

**Figure 3:**
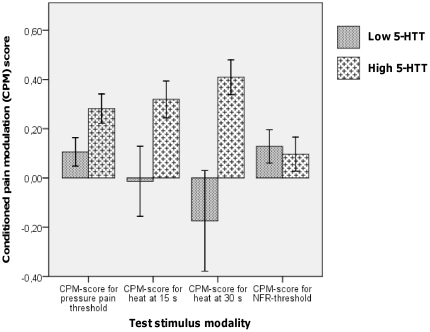
Conditioned pain modulation. The low 5-HTT-expressing group, as compared with the high 5-HTT group, had a significantly diminished conditioned pain modulation with regard to pressure pain thresholds [F(1,41) = 5.99, p = 0.02] and heat at 30 seconds [U = 145.5, z = −2.44, p = 0.02, r = −0.36]. There were no significant differences between groups with regard to tonic pain-mediated increase in the threshold for the nociceptive flexion reflex (NFR), F<1.

Genotype did not have a significant effect on the baseline PPTs [U = 247, z = −0.14, p = 0.90]. See Figure 2 A. As expected, men had significantly higher PPTs than women [U = 96.5, z = −3.31, p<0.001]. All subjects followed through with the PPT-testing during the SETT. A manipulation check for PPTs at baseline compared to those obtained during CPM-testing was significant [z = −3.57, p<0.001], i.e. PPTs rose significantly during tonic pain as expected. A significant main effect of tri-allelic 5-HTTLPR-genotype on PPT CPM-score was found [F(1,41) = 5.99, p = 0.02] such that the low 5-HTT-expressing group did not increase their individually normalized PPTs as much as the high-expressing group. In other words, the low 5-HTT-expressing group displayed reduced CPM-scores (0.11±0.27) for PPT when compared to the high 5-HTT-expressing group (0.28±0.29). See Figure 3. No effect of gender [F<1] or gender by genotype interactions [F(1,41) = 2.80, p = 0.101] were found. No effect of menstrual-cycle phase was found [U = 92, z = −0.25, p = 0.8].

### Heat-pain: ratings of suprathreshold noxious heat

**Figure 4:**
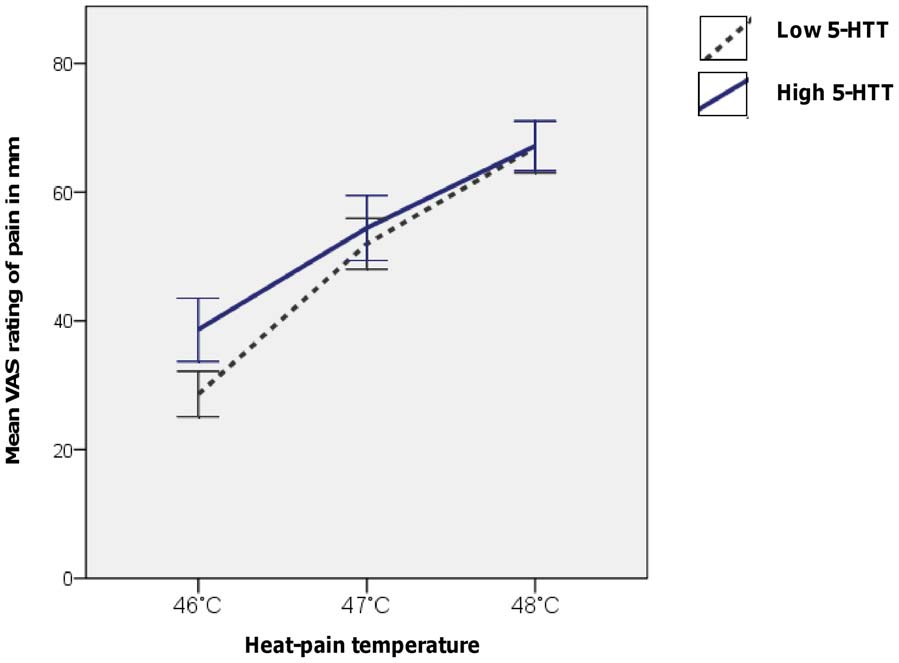
Interaction between pain-ratings for suprathreshold noxious heat and genotype. Mean VAS-ratings for suprathreshold noxious heat, applied to the right ventral forearm, are shown for the low 5-HTT-expressing and high 5-HTT-expressing genotype groups, respectively. The interaction between temperature level and genotype on the VAS-rating was significant [F(1.60, 65.51) = 4.10, p = 0.03].

The measurements obtained during the calibration exhibited the expected significant main effect of the temperature level on VAS- ratings [F(1.60, 65.51) = 150.92, p<0.001]. I.e. higher temperatures implied higher ratings of pain. At any given temperature, there were no significant differences in VAS -ratings between genotype groups – despite a tendency at 46.0°C for lower ratings in the low 5-HTT expressing group [t(39.7) = −1.65, p = 0.11 for the average of the two stimuli and t(40.5) =  −1.97, p = 0.06 for the first rating only]. However, a significant interaction between temperature level and genotype on the VAS-ratings emerged [F(1.60, 65.51) = 4.10, p = 0.03] such that the low 5-HTT-expressing group’s VAS-ratings increased significantly more with increasing temperatures as compared to the high 5-HTT-expressing group. See Figure 4. There was no significant interaction between gender and temperature on the VAS ratings, F<1.

### Heat-pain: baseline and during SETT-pain

No significant differences were found on the basis of genotype [U = 211, z = −0.95, p = 0.35] or gender [U = 209, z = −0.68, p = 0.51] between the temperatures used as test-stimuli. See Figure 2 B. For the individualized temperature, applied at baseline to the leg, no significant effect of time point on VAS -rating was found [z = −0.08, p = 0.94]. There were no significant differences between the high- versus low 5-HTT-expressing groups with regard to baseline heat-pain at 15 or 30 seconds, F<1 for both. The temperatures were intended to yield a 60 mm VAS-rating at baseline and achieved this with reasonable precision. The low 5-HTT-expressing group’s mean VAS-rating at 15 seconds was 46 mm±18.3 mm compared to the 5-HTT-expressing group’s mean rating of 47 mm±15.1 mm. The corresponding values at 30 seconds were 45 mm±22.0 mm and 47 mm±20.0 mm for the two groups, respectively. The aim of the calibration was not the exact level of the VAS-ratings, but rather to achieve a standardized baseline pain perception.

All subjects followed through with the heat-pain testing during the SETT. Individual heat-pain CPM-scores were calculated for the 15 and 30 second time points, respectively. To control for the familywise error due to this multiple testing, a p<0.025 was considered significant. At the 15 second time point genotype groups did not differ significantly, but exhibited a trend [U = 172.0, z = −1.84, p = 0.07]. At the 30 sec time point, however, the high (score = 0.41±0.34) and low (score = −0.18±0.96) 5-HTT-expressing group differed significantly in their CPM-scores, [U = 145.5, z = −2.44, p = 0.015, r = −0.36]. See Figure 3. No effect of menstrual-cycle phase was found on the measurement at 15 seconds [U = 62, z = −1.6, p = 0.1] or at 30 seconds [U = 84, z = −0.6, p = 0.6]. In the low 5-HTT-expressing group 7 individuals (3 women, 4 men) actually exhibited negative CPM (i.e. hyperalgesia) at 30 seconds compared to only 2 subjects in the high expressing group (one of each gender). Dichotomizing the 30 sec heat-pain data into negative versus positive CPM showed a non-significant trend with the low-expressing genotype group being more frequent in the negative CPM group [p = 0.07, Fisher’s exact test].

### Nociceptive flexion reflex

#### Number and timing of shocks

Across all trials, the average time between two shocks was 10 seconds (range 7 s–16 s). The number of shocks required for a threshold measurement ranged between 7 and 32 (mean = 13.8±4.6).

#### Baseline NFR-threshold values

Two consecutive baseline NFR-threshold measurements were conducted for each subject and the two threshold values were averaged for each individual. No significant differences for this average baseline NFR-threshold were found on the basis of genotype or gender, F<1 for both. See Figure 2 C.

#### NFR-threshold during the SETT-pain

Two subjects in the low 5-HTT-expressing group (one of each gender) interrupted the CPM-testing, due to the ischemic SETT-pain, before assessing the NFR- threshold. One additional male in the low 5-HTT-expressing group did not complete NFR-testing during the SETT, due to technical difficulties. All subjects in the high 5-HTT-expressing group completed the testing. The NFR-threshold rose significantly during the SETT [t(41) =  −2.03, p<0.05]. The average baseline threshold of 8.5 mA±4.7 mA rose during the ischemic pain to 9.2 mA±5.2 mA. No significant differences of CPM-scores with regard to gender, genotype or their interaction were found, F<1 for all. No effect of menstrual-cycle phase was found [U = 79, z = −0.54, p = 0.6]. See Figure 3.

### Correlations between CPM-scores for different modalities

The CPM-scores for PPTs displayed a high degree of correlation with the CPM-scores for heat-pain at 30 seconds [Spearman’s rho = 0.35, p = 0.02], a correlation that remained significant even after controlling for genotype [partial Spearman’s rho = 0.28, p = 0.03, one-tailed test]. However, neither values were significantly correlated with the CPM-scores of the NFR-thresholds [Spearman’s rho = −0.17, p = 0.27 for heat and rho = 0.03, p = 0.85 for PPT].

### Submaximal effort tourniquet test (SETT) parameters

#### Number of grips and grip-strength

Three subjects in each genotype group completed the maximum number of 60 grips (2 women and 1 male in each group). Number of grips needed ranged between 10 and 60 (median = 40). Genotype groups did not differ significantly in grip-strength [U = 242.5, z = −0.24, p = 0.82] or with regard to the number of grips needed, F<1.

#### Ischemic pain ratings during SETT

At the onset of CPM-testing during the SETT (i.e. at first verbal Borg-rating of 6 or above, or after the completion of 60-grips) the difference between genotype groups with regard to verbal ratings of ischemic pain was not significant [U = 189.5, z = −1.71, p = 0.08]. The mean of the two integer ratings for the ischemic pain obtained during CPM-testing was calculated for each subject. The low 5-HTT-expressing group exhibited a significantly higher mean for these pain ratings (7.7±1.0) as compared to the high-expressing group (6.9±1.9) [U = 167.5, z = −1.97, p<0.05].

### Potential influence of age on CPM

There may be some reason to believe that descending inhibition may deteriorate at or after middle-age [42].Genotype groups did not differ significantly in age and the majority of our subjects were in their twenties. See Table 1. It should therefore be noted that we have very limited statistical power (skewed age distribution with a high density around 25 years) to detect any such age-related CPM-variation. As an exploration, we calculated non-parametric correlations between age and our CPM-scores. No significant correlations emerged [rho for age vs PPT = −0.17, p = 0.26; rho for heat-pain at 15 s = 0.05, p = 0.73; rho for heat-pain at 30 s = 0.02, p = 0.9; rho for NFR = 0.11, p = 0.48].

## Discussion

Our main finding was that conditioned pain modulation (CPM) diverged on the basis of tri-allelic 5-HTTLPR in healthy individuals. Based on the *SLC6A4* gene’s putative involvement in chronic pain states such as fibromyalgia [28] and the fact that such conditions may be accompanied by experimental deficits in pain regulation [12] we hypothesized that the gene would also be associated with CPM-response in healthy individuals. We demonstrate a strong effect of genotype. The low 5-HTT-expressing group, as compared to high 5-HTT-expressing, experienced a significantly lower degree of CPM-mediated inhibition of pressure- and heat-pain sensitivity. The NFR-thresholds increased significantly during the application of tonic ischemic pain, indicating the presence of the expected CPM-mediated inhibitory effect on spinal nocifensive reflexes. Interestingly, however, there were no significant differences between the genotype groups with regard to this modulation.

Our findings are at variance with a recent study that failed to find a relationship between the 5-HTTLPR and CPM [43]. The discrepancy may be explained by differences in statistical power as well as methodologies. Potvin et al stratified for the bi-allelic 5-HTTLPR, without additionally considering the rs25531. There is good evidence that the mini-haplotype (5-HTTLPR, rs22531) confers additional resolution to a study as the G-allele, on the 5-HTTLPR l-allele background, reduces the transcriptional efficacy to the level of the short-allele [44]. A perhaps more important factor, however, in explaining the discordant findings is the sensitivity of the actual CPM-paradigm. A review recently concluded that the application of test-stimuli in parallel with the conditioning pain, as in our study, yields higher CPM-mediated inhibition compared to sequential paradigms [45] as used by Potvin et al. Nonetheless, Potvin and colleagues did successfully replicate previous results [12] showing an impaired pain modulation in fibromyalgia patients.

It has been suggested that the degree of pain experienced due to the conditioning stimulus may be positively correlated to the degree of pain inhibition through CPM [10]. This supports our conclusion that the observed differences represent a substantive finding since the low 5-HTT-expressing group experienced a significantly reduced CPM-response despite reporting a significantly higher level of ischemic pain during the SETT, compared to the high 5-HTT-expressing group. Interestingly, a reduced tolerance to ischemic pain has been reported in depressed individuals [46], [47]. Although our subjects were healthy and non-depressed, low 5-HTT-expression is a known risk-factor for depression [48] and our results may hence hint at a potential role of 5-HTT-related mechanisms in ischemic pain-sensitivity.

A possible interpretation of our finding may be that it represents a relative shift along the balance between nociceptive inhibition and facilitation, both engaged during tonic ischemic muscle pain, towards the latter in the low 5-HTT-expressing group. Muscle pain in myalgia and fibromyalgia patients reduces descending pain inhibition during physical activity, stressing the clinical relevance of these mechanisms [49]. Similarly, in healthy individuals, experimental muscle pain has been shown to impair descending inhibition when applied together with a cold-pressor test [50]. In our experiment, peripheral differences in 5-HT metabolism may have influenced such pro-nociceptive facilitation during the ischemia. The 5-HTT regulates serotonin plasma levels through uptake into platelets [51] with higher transporter expression implying lower levels of serotonin in the blood [52]. Peripheral 5-HT receptors have been implicated in muscle pain. Accordingly, intramuscular injections of, granisetron, a 5-HT_3_ receptor antagonist, have been shown to reduce experimental muscle pain [53].

Interestingly, our baseline calibration of suprathreshold heat-pain temperatures revealed an interaction between genotype and pain rating. The low 5-HTT-expressing group’s pain ratings increased significantly more than the high 5-HTT-expressing group with increasing temperature. Taken together with the results for the CPM-test, this may imply that either increasing the stimulus intensity or adding a concomitant tonic pain might induce pro-nociceptive facilitation in the low 5-HTT-expressing group.

In the initial electrophysiological reports of DNIC-phenomena in rodents, Le Bars and colleagues contend that “DNIC seems to be to a great extent dependent on the integrity of the descending serotonergic system.” [8]. Pharmacological studies point towards the same conclusion [17],[54]. Resembling the work done on animal models, the presence of CPM in humans was initially investigated using the nociceptive flexion reflex (NFR). It was found that the threshold for the reflex was linked to the subjective report of pain elicited by the NFR-testing itself, and that the threshold of both increased after heterotopically applying a conditioning pain [10], [55].

The NFR has been used in many clinical studies and employed as a research tool for studies of acute and chronic pain [34]. As expected, we found a significant upward shift in NFR-thresholds during tonic pain, but without any differences between the groups. Whereas the CPM-score for the reported pain experience of pressure and heat- pain where strongly correlated, there was not even a tendency for correlation between the shifts in ratings of subjective pain and the shift in NFR-threshold. Comparing different reports, the ‘subjective studies’ and ‘objective studies’ seem to show equal effect sizes [45]. To the best of our knowledge, though, this is the first report of CPM-effects determined simultaneously for NFR along with other test-modalities in the same subjects. Importantly, however, it is not the first time subjective pain reports have been decoupled from the NFR-response [56], [57], [58].

With a CPM related to different levels of inferred 5-HTT-expression, we have the opportunity to compare reported pain experience with a neurophysiological measurement of spinal nocifensive processing in relation to serotonin metabolism. Obviously pain perception is highly dependent on cortical mechanisms. As mentioned, we found a decoupling of the CPM-score for spinal nocifensive reflex activity from CPM-scores based on subjective pain report. Neuroreceptor imaging in healthy human subjects has shown an increase in selective binding to supraspinal 5-HTT in subjects homozygous for the L_A_ allele [59]. In the field of affective regulation, numerous differences have been found with regard to the 5-HTTLPR [23]. The low 5-HTT-expressing group reported a slight but significant higher state-anxiety compared to the high-expressing group. Such differences may have altered the capacity to recruit descending inhibitory systems during tonic pain as well as influenced the cortical processing of the pain percepts per se.

Another possible interpretation of the decoupling between NFR-thresholds and subjective pain during CPM is that, whereas flexor responses appear to rely on wide-dynamic range (WDR) neurons in deeper lamina (e.g. lamina V) [60], the actual perception of heat and pressure pain intensity may depend more on afferent activity reaching nociceptive specific (NS) neurons in lamina I [61]. Lamina I neurons are known to project to areas of the insular cortex [62], an important interoceptive area, the functioning of which, in turn, has been demonstrated to be directly affected by SSRIs [63].

The NFR is part of a spinal network of interneurons onto which many so-called flexor-reflex-afferents (FRAs) of varied, but mainly non-nociceptive, peripheral origin synapse [35]. Despite the evidence for the important role of 5-HT in DNIC-effects observed in deeper WDR neurons, and the complex interplay between WDR and NS neurons [64], there is nothing that would rule out differential serotonergic modulation of lamina I neurons. Although deeper lamina are rich in 5-HT fibers, lamina I and II are the most abundant in varicosities [65] and 5-HTT has been shown to co-localize with such varicosities in the spinal cord [66]. Also, lamina I neurons are almost unique in receiving direct projections from the hypothalamus which have been shown to produce antinociceptive effects mediated through 5-HT_1A_, 5-HT_1B_ and 5-HT_3_ receptors [61], [67]. The superficial laminae are also densely innervated by descending serotonergic neurons from the rostroventral medulla (RVM) from which certain neuronal populations- illustrating the complexity of spinal nociceptive processing – have a facilitatory role. It should however be emphasized that such facilitation may be more involved in the development of persistent pain rather than in the perception of acute pain [68].

The individual classes of serotonin receptors exhibit a myriad of reported interactions. For example, the superficial laminae of the dorsal horn are rich in 5-HT_1A_ receptors which can partake in both inhibitory and facilitory processes [69]. The density of receptors is dynamic and 5-HTT knockout models illustrate the compensatory up-regulation (of e.g. 5-HT_3_) as well as functional down- regulation of others (e.g. 5-HT_1A_) [70]. We recently reported that low 5-HTT-expressing individuals experience greater pain relief than high 5-HTT-expressing individuals after an injection of the short-acting opioid remifentanil and suggested how this may be linked to a desensitization of 5-HT_1A_ receptors [71]. While 5-HT_1A_ receptor agonists may promote pronociception at baseline, possibly explaining the observed interaction between genotype and suprathreshold heat-pain ratings, 5-HT_1A_ receptor activation promotes analgesia during concurrent tonic pain stimulation[72], [73]. This would seem to fit well with our current results, with putatively down-regulated 5-HT_1A_ receptors in the low 5-HTT-expressing group which indeed exhibited a reduced pain inhibition during tonic pain.

It is likely that a number of the discussed mechanisms are at play in the demonstrated genotypic differences. Our study had several limitations and conclusions drawn from studies using evoked pain of short duration in healthy volunteers cannot necessarily be extrapolated to the clinical setting. 5-HTT expression was inferred from genotype rather than measured directly and, as in all genetic-associations studies, causality cannot be asserted. For further interpretation, our findings need to be replicated for larger samples of individuals in studies aimed at teasing apart the involved mechanisms. As the ability to inhibit pain through CPM has been prospectively tied to risk of developing chronic post-surgical pain [16], it would therefore be of great clinical interest to assess any effects of tri-allelic 5-HTTLPR on the chronification of acute pain. This could potentially help to identify individuals at risk and, if coupled to a greater mechanistic understanding of the underlying pain processes, may direct preemptive pharmacological treatment.

In sum, our results demonstrate the involvement of the *SLC6A4* gene in explaining aspects of clinically relevant individual differences in pain perception and regulation. The tri-allelic 5-HTTLPR appears to be associated with healthy European adults’ capacity to recruit pain modulatory systems in an experimental setting of acute tonic ischemic pain. Furthermore, our results illustrate that shifts in NFR-thresholds do not necessarily correlate to the modulation of experienced pain. The differences in reported pain experience may represent a shift in the balance between inhibition and facilitation, towards the latter, in the group with low 5-HTT-expression. Additionally, cortical factors related to the pain perception per se may have differed between groups, as perhaps suggested by the differences in anxiety levels. The involved mechanisms may be related to the putative up/down -regulation of various receptors, e.g. 5-HT_1A_, along different levels of the neuraxis as a function of differential 5-HTT expression.
